# Vitamin D and Vitamin D Binding Protein in Health and Disease 2.0

**DOI:** 10.3390/ijms241210316

**Published:** 2023-06-19

**Authors:** Charlotte Delrue, Marijn M. Speeckaert

**Affiliations:** 1 Department of Nephrology, Ghent University Hospital, 9000 Ghent, Belgium; charlotte.delrue@ugent.be; 2 Research Foundation-Flanders (FWO), 1000 Brussels, Belgium

Vitamin D, often referred to as the “sunshine nutrient”, has gained considerable attention in recent years due to its multifaceted impact on health and disease. In the Kandutsch–Russel pathway, 7-dehydrocholestrol (7DHC) serves as an immediate precursor to cholesterol. It can absorb ultraviolet B radiation (UVB) and convert it into pre-vitamin D_3_. Pre-vitamin D_3_ can undergo isomerization to form vitamin D_3_. Prolonged exposure to UVB can also transform it into lumisterol 3 (L3) and tachysterol 3. The enzymes 25-hydroxylase (CYP2R1 or CYP27A1) and 1α-hydroxylase (CYP27B1) activate vitamin D_3_ in the canonical pathway. This activation process leads to the production of 1,25(OH)_2_D_3_. Both 25(OH)D_3_ and 1,25(OH)_2_D_3_ can undergo degradation through sequential oxidation of the side chain, catalyzed by CYP24A1. For 1,25(OH)_2_D_3_, this degradation results in the formation of calcitroic acid. A similar series of reactions occur for the activation of vitamin D_2_, which is formed when fungi- or phytoplankton-derived ergosterol undergoes transformation induced by UVB. This process involves the same enzymes and pathways as vitamin D_3_. In addition, there is a novel non-canonical pathway for the activation of vitamin D_3_. This pathway begins with CYP11A1 initiating hydroxylation at either the C20 or C22 position. Subsequent hydroxylation occurs at C20, C22, C23, and/or C17. The resulting products can then undergo selective hydroxylation by enzymes such as CYP2R1, CYP3A4, CYP24A1 and/or CYP27A1. All intermediates, except those with a hydroxyl group at C17, can undergo additional hydroxylation at C1a. CYP11A1 can also hydroxylate vitamin D_2_, resulting in the formation of 20(OH)D_2_, 17,20(OH)_2_D_2_, and 17,20,24(OH)_3_D_2_. Moreover, 20(OH)D_3_ can be hydroxylated by CYP27B1, leading to the production of 1,20(OH)_2_D_2_. Finally, 7DHC and L3 are also substrates for CYP11A1. 7DHC undergoes conversion into 22(OH)7DHC, 20,22(OH)27DHC, and 7-dehydroepregnenolone. In contrast, L3 produces 20(OH)L3, 22(OH)L3, 20,22(OH)2L3, 24(OH)L3, and pregnalumisterol through the action of CYP11A1 [1].

The conventional perspective suggests that most of the diverse effects of vitamin D are mediated through the interaction between 1,25(OH)_2_D_3_ and the nuclear vitamin D receptor (VDR). Once bound to 1,25(OH)_2_D_3_, the receptor forms a heterodimer with the retinoid X receptor (RXR) and is translocated to the nucleus. It binds to the VDR-responsive element (VDRE) present in target genes, thereby influencing their expression and regulation. Furthermore, it has been postulated that the actions of 1,25(OH)_2_D_3_ include non-genomic activities that are associated with its interaction with 1,25D_3_-MARRS (ERp57 or PDIA3). This protein is located in the endoplasmic membrane and functions as a thiol-disulfide oxidoreductase. Additionally, it plays a role in the folding and quality control of newly synthesized glycoproteins. Thus, the non-genomic activity of 1,25(OH)_2_D_3_ contributes to its overall effects [1].

Novel vitamin D_3_-hydroxyderivatives derived from CYP11A1 have demonstrated significant anti-proliferative, pro-differentiation, and anti-inflammatory effects in vitro, which are comparable to or even superior to those of 1,25(OH)_2_D_3_. Moreover, alternative nuclear receptors have been identified for these CYP11A1-derived D_3_-hydroxyderivatives, and to some extent, for classical 1,25(OH)_2_D_3_ as well. These receptors include retinoid-related orphan receptors (ROR)α and γ and the arylhydrocarbon receptor (AhR), which function as inverse agonists and agonists, respectively. The binding and activation of these receptors by different vitamin D_3_-derivatives, including 1,25(OH)_2_D_3_, are dependent on the structural localization of the hydroxyl groups. This localization determines the specificity and affinity of the vitamin D_3_-derivatives for genomic and non-genomic binding sites associated with the VDR, ROR α and γ, AhR, and potentially, 1,25D_3_-MARRS [1].

The present Special Issue “Vitamin D and Vitamin D Binding Protein in Health and Disease 2.0” presents a collection of papers that explore the implications of vitamin D and vitamin D binding protein across diverse domains. These studies not only contribute to our understanding of the intricate workings of this essential nutrient but also pave the way for future research and potential breakthroughs in healthcare. The first, a thought-provoking paper titled “Vitamin D Deficiency: An Underestimated Factor in Sepsis?”, raises awareness of the potential link between vitamin D deficiency and sepsis, a potentially fatal condition with high mortality rates [2]. This study highlights the need for further research to explore the effect of vitamin D supplementation on sepsis outcomes, potentially leading to novel therapeutic interventions. Unraveling the underlying mechanisms and developing effective interventions could have a transformative impact on sepsis management and patient outcomes. Continuing the exploration of the role of vitamin D in infectious diseases, a subsequent paper titled, “A Simultaneous Extraction/Derivatization Strategy for Quantitation of Vitamin D in Dried Blood Spots Using LC-MS/MS: Application to Biomarker Study in Subjects Tested for SARS-CoV-2”, introduces a ground-breaking tool for precisely determining vitamin D concentrations in dried blood spots [3]. The study was able to precisely measure 25(OH)D_3_, vitamin D_3_, 25(OH)D_2_, and vitamin D_2_ in human blood using this method with no discernible matrix effects in subjects who had been tested for SARS-CoV-2. The outcomes showed reasonable accuracy and precision, conforming to FDA standards. This research opens doors for further investigation into the interplay between vitamin D, infectious diseases, and immune function.

Addressing the complex interplay between vitamin D and thyroid function, “Vitamin D and the Thyroid: A Critical Review of the Current Evidence” critically evaluates the existing literature to shed light on the potential implications of the vitamin D status in thyroid disorders [4]. This comprehensive review provides a thorough analysis of the available evidence, highlighting the knowledge gaps and areas for further investigation. Understanding the complex relationship between vitamin D and thyroid health may lead to the development of targeted interventions and personalized treatment approaches in the future. Expanding on the exploration of the vitamin D–thyroid connection, a study titled “Vitamin D Status in Patients before Thyroidectomy” shows the high prevalence of a significant vitamin D deficiency and the proper concentration of 1,25(OH)_2_D before thyroid surgery [5]. These findings highlight the importance of the determination of vitamin D concentrations prior to thyroidectomy to prevent hypocalcemia.

While the specific mechanisms are still being elucidated, it is plausible that optimizing vitamin D levels may have beneficial effects on both weight management and blood pressure control. The intriguing paper “Vitamin D and Weight Change: A Mendelian Randomization, Prospective Study” explores the causal relationship between vitamin D and weight regulation [6]. Using a powerful genetic analysis technique, this study demonstrates a little association between genetically determined vitamin D levels and weight or waist gain. Understanding the interplay between vitamin D and weight change opens new avenues for innovative strategies to combat obesity and its associated health complications, potentially leading to improved public health outcomes. Furthermore, this Special Issue also includes a comprehensive review titled “Latest Knowledge on the Role of Vitamin D in Hypertension” [7]. This study synthesizes the most recent evidence on the relationship between vitamin D and hypertension, a major global health concern. By highlighting the challenges in terms of proving a direct antihypertensive effect of vitamin D on its own, this research emphasizes the need for novel studies with higher doses of vitamin D, larger populations, and longer treatment periods to evaluate the potential of vitamin D supplementation as a preventive and therapeutic approach to manage hypertension.

Understanding the relationship between vitamin D, chronic liver diseases, and autoimmune diseases may provide insights into shared immune-related mechanisms and potential therapeutic strategies. “The Role of Vitamin D and Vitamin D Binding Protein in Chronic Liver Diseases” investigates the relationship between vitamin D, its binding protein, and chronic liver diseases [8]. This research not only deepens our understanding of the role of vitamin D and vitamin D binding protein in liver health but also paves the way for potential interventions to improve patient outcomes and prevent disease progression. Further studies in this area could explore the efficacy of vitamin D-based interventions in ameliorating liver disease and optimizing liver function. Delving into the complex interplay between vitamin D and the immune system, particularly in the context of autoimmune diseases, the paper titled “An Update on the Effects of Vitamin D on the Immune System and Autoimmune Diseases” provides useful insights [9]. By reviewing the latest research, this study offers a comprehensive understanding of the immunomodulatory effects of vitamin D and its potential role in the prevention and management of psoriasis, type 1 diabetes, multiple sclerosis, inflammatory bowel diseases, rheumatoid arthritis, systemic lupus erythematosus, systemic sclerosis, etc. This knowledge not only deepens our understanding of the intricate interplay between vitamin D and immune function but also highlights the potential of vitamin D-based interventions in the management of autoimmune diseases.

In conclusion, the Special Issue on “Vitamin D and Vitamin D Binding Protein in Health and Disease 2.0” has offered a thorough examination of the multifaceted role of vitamin D in various aspects of health and disease. The collection of papers sheds light on the impact of vitamin D on the immune response, infectious diseases, thyroid health, weight regulation, hypertension, autoimmune diseases, and chronic liver diseases. The findings from these studies have significantly advanced our understanding of the interplay between vitamin D, its binding protein, and various physiological processes. The methodologies developed in this Special Issue, such as the simultaneous extraction/derivatization strategy for quantifying vitamin D in dried blood spots and the Mendelian randomization approach for studying causal relationships, have introduced innovative techniques that enhance our ability to assess and analyze the effects of vitamin D in clinical and research settings. These advancements will pave the way for future studies and interventions aimed at optimizing vitamin D levels and leveraging their potential health benefits.
